# Molecular identification and histopathological study of natural *Streptococcus agalactiae* infection in hybrid tilapia (*Oreochromis niloticus*)

**DOI:** 10.14202/vetworld.2017.101-111

**Published:** 2017-01-24

**Authors:** AA Laith, Mohd Azmi Ambak, Marina Hassan, Shahreza Md. Sheriff, Musa Nadirah, Ahmad Shuhaimi Draman, Wahidah Wahab, Wan Nurhafizah Wan Ibrahim, Alia Syafiqah Aznan, Amina Jabar, Musa Najiah

**Affiliations:** 1 School of Fisheries and Aquaculture Sciences, Universiti Malaysia Terengganu, 21030 Kuala Terengganu, Malaysia; 2 Institute of Tropical Aquaculture (AQUATROP), Universiti Malaysia Terengganu, 21030 Kuala Terengganu, Malaysia; 3 Department of Microbiology, Zhejiang University School of Medicine, 866 YuHuaTang Lu, Hangzhou, Zhejiang, China

**Keywords:** 16S rDNA, antibiotic resistance, aquaculture, histopathological examination, polymerase chain reaction, *Streptococcus agalactiae*

## Abstract

**Aim:**

The main objective of this study was to emphasize on histopathological examinations and molecular identification of *Streptococcus agalactiae* isolated from natural infections in hybrid tilapia (*Oreochromis niloticus*) in Temerloh Pahang, Malaysia, as well as to determine the susceptibility of the pathogen strains to various currently available antimicrobial agents.

**Materials and Methods:**

The diseased fishes were observed for variable clinical signs including fin hemorrhages, alterations in behavior associated with erratic swimming, exophthalmia, and mortality. Tissue samples from the eyes, brain, kidney, liver, and spleen were taken for bacterial isolation. Identification of *S. agalactiae* was screened by biochemical methods and confirmed by VITEK 2 and 16S rRNA gene sequencing. The antibiogram profiling of the isolate was tested against 18 standard antibiotics included nitrofurantoin, flumequine, florfenicol, amoxylin, doxycycline, oleandomycin, tetracycline, ampicillin, lincomycin, colistin sulfate, oxolinic acid, novobiocin, spiramycin, erythromycin, fosfomycin, neomycin, gentamycin, and polymyxin B. The histopathological analysis of eyes, brain, liver, kidney, and spleen was observed for abnormalities related to *S. agalactiae* infection.

**Results:**

The suspected colonies of *S. agalactiae* identified by biochemical methods was observed as Gram-positive chained cocci, β-hemolytic, and non-motile. The isolate was confirmed as *S. agalactiae* by VITEK 2 (99% similarity), reconfirmed by 16S rRNA gene sequencing (99% similarity) and deposited in GenBank with accession no. KT869025. The isolate was observed to be resistance to neomycin and gentamicin. The most consistent gross findings were marked hemorrhages, erosions of caudal fin, and exophthalmos. Microscopic examination confirmed the presence of marked congestion and infiltration of inflammatory cell in the eye, brain, kidney, liver, and spleen. Eye samples showed damage of the lens capsule, hyperemic and hemorrhagic choroid tissue, and retina hyperplasia accompanied with edema. Brain samples showed perivascular and pericellular edema and hemorrhages of the meninges. Kidney samples showed hemorrhage and thrombosis in the glomeruli and tubules along with atrophy in hematopoietic tissue. Liver samples showed congestion of the sinusoids and blood vessel, thrombosis of portal blood vessel, and vacuolar (fatty) degeneration of hepatocytes. Spleen samples showed large thrombus in the splenic blood vessel, multifocal hemosiderin deposition, congestion of blood vessels, and multifocal infiltration of macrophages.

**Conclusion:**

Therefore, it can be concluded that pathological changes in tissues and organs of fish occur proportionally to the pathogen invasion, and because of their high resistance, neomycin and gentamicin utilization in the prophylaxis or treatment of *S. agalactiae* infection should be avoided.

## Introduction

Tilapia (*Oreochromis niloticus*) is described to be among the top cultured fish worldwide including Malaysia [1]. *Streptococcus agalactiae* is a significant human and animal pathogen [2] with strong association to a variety of diseases, most importantly Streptococcosis, which is a highly problematic disease in the aquaculture of freshwater fishes, constantly threatening the sustainability of the global tilapia industry [3]. Periodically, this pathogen has been linked to illness in many others hosts including chickens, camels, dogs, horses, cats, frogs, hamsters, mice, and monkeys. Therefore, not only does *S. agalactiae* compromise food safety but also represent a zoonotic hazard [4].

*S. agalactiae* infection in tilapia caused a variety of clinical signs to the infected fish. The most common clinical signs of *S. agalactiae* infections in tilapia include erratic swimming (spiraling or spinning), unilateral or bilateral exophthalmia (pop-eye), corneal opacity, eye hemorrhage, and hemorrhages at the base of the fins and opercula. Darkening of the skin, distended abdomen and body curvature, or vertebral deformities have been observed [5-7]. Internally, abnormalities are detected and are grossly visible in the liver, spleen, kidney, eyes, and brain. The affected organs showed enlargement, hemorrhages, inflammation, and ascites [8,9]. On the other hand, several affected fish did show any clinical signs before sudden death [10,11].

*Streptococcus* spp. is associated with fatal outcomes in tilapia and is the most important fish pathogen in Malaysia today [1]. During the last several years, there have been copious *S. agalactiae* infection outbreaks and were documented in numerous farms [12,13]. *S. agalactiae* has a mortality rate of 70% in red tilapia (*O. niloticus* hybrid) in cages of Kenyir, Pedu, and Pergau Lakes [1,6]. Unfortunately, little information is available describing the evolution of histological lesions in Tilapia infected naturally by *S. agalactiae*. Thus, this study was conducted for the purpose of isolation and identification of the most prevalent bacteria causing septicemic diseases in cage-cultured fresh-water fishes in several private farms in Temerloh Pahang, Malaysia; in addition, to try and decrease the economic losses caused by such disease causing pathogens by applying preventative and treatment measures using a drug of choice depending on culture sensitivity test. Therefore, we believe this new work shows veterinarians the events of natural *S. agalactiae* infection in Tilapia and is helpful in reaching a correct rapid diagnosis whereby they can follow our guide in isolation and identification and the proper application of medicine to face economic losses. Finally, this research project will also help researchers to compare or confirm our result, especially with histopathology data.

## Material and Methods

### Ethical approval

This study was carried out in accordance with the guidelines of the International Animal Ethics Committee and is in accordance with local laws and regulations. Adequate measures were taken to minimize discomfort of our animal subjects.

### Experimental fish

Ten apparently naturally infected hybrid tilapia (*O. niloticus*) weighing 200-300 g were collected from farm ponds in Temerloh Pahang Province, Malaysia. Then, the disease signs were observed and recorded. 10 fish with clinical signs were transferred alive in plastic bags with an oxygen supply to the Fish Health Laboratory (AQUATROP) in University Malaysia Terengganu for further study. The fish was anesthetized with Tricaine Methanesulfonate (MS-222), dissected [14] and submitted to autopsy.

### Bacterial isolates

Samples were taken for routine bacteriological examination from; eye, brain, liver, spleen, and kidney of hybrid tilapia (*O. niloticus*). They were then inoculated onto brain heart infusion (BHI) agar (Merck, Germany) and incubated at 30°C for 24 h. The dominant colonies were subcultured on the same media to check the purity of the isolate. After incubation at 30°C for 24 h, bacterial colonies were picked and plated on blood agar (MERCK, Germany) plates until pure cultures were obtained. Pure stock isolates were stored at −20°C in 15% glycerol (final concentration) supplied with BHI broth.

### Biochemical characteristics of the isolates

Biochemical characteristics of the isolates were confirmed by microbial biochemical identification basis of standard phenotypic testing criteria, Gram-stain, motility, oxidase activity, growth characteristics, and hemolysis test. The phenotypic systems examined in this study using the VITEK 2 Systems Version: 5.04 ID card (BioMérieux, Inc., Hazelwood, MO) with reference to Berger’s Manual of Determinative Bacteriology [15].

### Growth characteristics of the isolates

Growth characteristics of the isolates performed with minor modifications according to Buller [16].

### Effect of temperature

About 25 µl of fresh bacterial suspension was inoculated into test tubes filled with 5 ml sterile on BHI broth (MERCK, Germany) and incubated at 5, 10, 15, 25, 30, 35, 40 and 45°C. 10 tubes were maintained at each temperature post-inoculation. After 18 h, all the cultures were sampled, and OD_610_ values were determined.

### Effect of pH

The pH range of sterile nutrient broth was adjusted to 3, 4, 5, 6, 7, 8, 9, 10 and 11. For each PH, 10 tubes filled with 5 ml sterile on BHI broth (MERCK, Germany) were prepared, inoculated with 25 µl of fresh bacterial suspension and incubated at 30°C. After 18 h, all the cultures were sampled, and OD_610_ values were determined.

### Effect of salinity

The salinity range of sterile on BHI broth (MERCK, Germany) was adjusted to 0%, 0.5%, 1%, 1.5%, 2%, 2.5%, 3%, 3.5%, 4%, 4.5%, 5%, 5.5% and 6% NaCl (w/v) for each salinity, 10 tubes filled with 5 ml sterile nutrient broth were prepared, and 25 µl of fresh bacterial suspension was inoculated into them. They were statically incubated at 30°C, and after 18 h, all the cultures were sampled, and OD_610_ values were determined.

### Molecular approaches

The identified *S. agalactiae* from the eye was subjected to 16S rRNA gene polymerase chain reaction (PCR) amplification by universal primers for confirmation of *S. agalactiae* [17].

### Bacterial culture and DNA extraction

The isolate was cultured in 3 ml of Tryptic soya broth (TSB) (MERCK, Germany) overnight at 37°C. The bacterial culture was centrifuged (14,000 ×*g* for 5 min at room temperature), pellet was harvested, and total genomic DNA (gDNA) of the isolates was extracted using Wizard^®^ gDNA Purification Kit, A1120 (Promega, USA) according to manufacturer’s protocol for gram positive bacteria. The extracted DNA was used as the template for PCR [18].

### PCR

The PCR reaction mixture of 16s rRNA was done in 25 µl total reaction using ×2 MyTaq mix (Bioline, UK) with 10 µM of each primer. Negative control served as non-template mixture [19]. The gDNA of the isolate was amplified for 16S rRNA by bacterial universal primers 8F (5’-GTTTACCTTGTTACGACTT-3’) and 1492R (5’-AGAGTTTGATCCTGGATGCTCAG-3’). The PCR reaction was performed in a Biothermal cycler (Bio-Rad, USA) with an initial denaturing step at 95°C for 5 min; 26 cycles of 95°C for 30 s, 55°C for 1 min and 72°C for 2 min; followed by 72°C for 10 min. 6 µl of the amplified products were electrophoresed by 1.2% (w/v) agarose gel in ×1 TBE electrophoresis buffer. Standard DNA ladder, 1 and 100 bp (Invitrogen, Germany) were used to confirm the size of the amplified PCR products at 1500 bp. The gel was stained with ethidium bromide (Promega, USA) and documented by UV-transilluminator (Bio-Rad, USA). Sequences obtained were analyzed and compared with sequences from GenBank using BLAST NCBI citation (http://blast.ncbi.nlm.nih.gov). The accession number of the *S. agalactiae* was deposited in GenBank.

### Antibiotic susceptibility

Antimicrobial susceptibility test was conducted to further understand the exposure of the strain toward antibiotics by standard antibiotics disc and disc diffusion technique [20]. A suspension (100 µl) of fresh cultured of *S. agalactiae* <24 h on TSB, diluted to a turbidity equivalent to a MacFarland No. 0.5 standard solution, was spread onto triplicate Mueller–Hinton agar (Oxoid, England) plates, and tested against 18 chemotherapeutic agent discs namely nitrofurantoin (F - 50 µg), flumequine (UB - 30 µg), florfenicol (FFC - 30 µg), amoxylin (AML - 25 µg), doxycycline (DO - 30 µg), oleandomycin (OL - 15 µg), tetracycline (TE - 30 µg), ampicillin (AMP - 10 µg), lincomycin (MY - 15 µg), colistin sulfate (CT - 25 µg), oxolinic acid (OA - 2 µg), novobiocin (NV - 30 µg), spiramycin (SP - 100 µg), erythromycin (E - 15 µg), fosfomycin (FOS - 50 µg), neomycin (N - 10 µg), gentamicin (GM - 10 µg), and polymyxin B (PB - 30 µg) (Oxoid, England). The susceptibility test agars were incubated at 37°C for 24 h and the diameter of inhibition zones around the discs was measured and compared to the standard table for antimicrobial susceptibility provided by CLSI [21].

### Histopathological examination

This is an outbreak case with high mortality in private farms in Temerloh Pahang, Malaysia, and all specimens were infected after inspection. Five samples of moribund fish were randomly selected to the laboratory. The fish was quickly euthanized with (MS-222, 60 mg/L) dissected and selected organs including liver, spleen, kidney, eye, and brain immediately fixed in 10% (v/v) neutral buffered formalin for histopathological examination. Tissues were dehydrated in an ethanol series, infiltrated and embedded in paraffin wax and sectioned on a rotary microtome at 5 µm. Sections were stained by hematoxylin and eosin and examined under advanced microscope [22].

## Results

### Morphologic and biochemical characteristics

The isolates showed to be Gram-positive cocci bacteria. After incubation on BHI agar (MERCK, Germany) at 30°C for 24 h, the colonies appeared to be raised and glossy, with a diameter of 1.5-2.0 mm. Biochemical characteristics of the isolates were consistent with *S. agalactiae* (Tables-1 and 2).

**Table-1:** Biochemical characteristics of *S.agalactiae* isolated from naturally infected hybrid tilapia (*Oreochromis* spp).

Characteristics	Result
Gram-stain	Positive
Shape	Coccus
Motility	Negative
Oxidase	Negative
Catalase	Negative
Starch	Negative
Lactose	Negative
Esculin	Negative
Glucose	Positive
Blood hemolysis	β-hemolytic

*S. agalactiae*=*Streptococcus agalactiae*

**Table-2:** Biochemical characteristics of suspected isolated from naturally infected hybrid tilapia (*Oreochromis* spp.) using Vitek 2 with 99% probability of *S. agalactiae*.

Well	Biochemical test	Result
2	D-amygdalin (AMY)	−
4	Phosphatidylinositol phospholipase C (PIPLC)	−
5	D-xylose (dXYL)	−
8	Arginine dihydrolase 1 (ADH1)	+
9	Beta-galactosidase (BGAL)	−
11	Alpha-glucosidase (AGLU)	−
13	Ala-Phe-Pro arylamidase (APPA)	−
14	Cyclodextrin (CDEX)	−
15	L-aspartate arylamidase (AspA)	−
16	Beta galactopyranosidase (BGAR)	−
17	Alha-mannosidase (AMAN)	−
19	Phosphatase (PHOS)	+
20	Leucine arylamidase (LeuA)	+
23	L-proline arylamidase (ProA)	−
24	Beta glucuronidase (BGURr)	−
25	Alpha-galactosidase (AGAL)	−
26	L-pyrrolidonyl-arylamidase (PyrA)	−
27	Betaglucuronidase (BGUR)	−
28	Alanine arylamidase (AlaA)	−
29	Tyrosine arylamidase (TyrA)	−
30	D-sorbitol (dSOR)	−
31	Urease (URE)	−
32	Polymixin b resistance (POLYB)	+
37	D-galactose (dGAL)	+
38	D-ribose (dRIB)	+
39	L-lactate alkalinization (ILATk)	−
42	L-actose (LAC)	−
44	N-acetyl-d-glucosamine (NAG)	+
45	D-maltose (dMAL)	+
46	Bacitracin resistance (BACI)	+
47	Novobiocin resistance (NOVO)	+
50	Growth in 6.5% NaCl (NC 6.5)	+
52	D-mannitol (dMAN)	−
53	D-mannose (dMNE)	+
54	Methyl-B-D-glucopyranoside (MBdG)	+
56	Pullulan (PUL)	−
57	D-raffinose (dRAF)	−
58	O/129 resistance (comp. vibrio) (O129R)	−
59	Salicin (SAL)	−
60	Saccharose/sucrose (SAC)	+
62	D-trehalose (dTRE)	+
63	Arginine dihydrolase 2 (ADH2s)	+
64	Optochin resistance (OPTO)	+

*S. agalactiae*=*Streptococcus agalactiae*

### Growth characteristics

**Figure-1:**
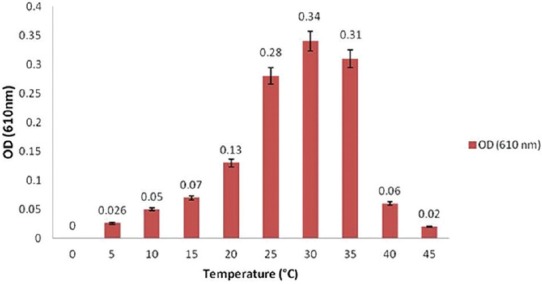
Effect of temperature on the growth of *Streptococcus agalactiae*.

**Figure-2:**
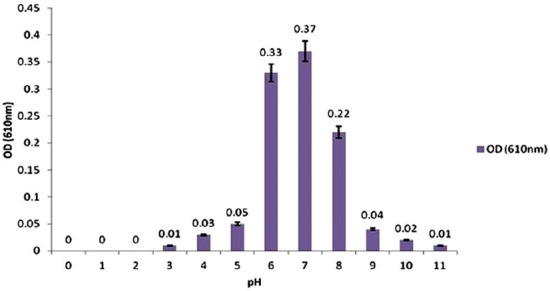
Effect of pH on the growth of *Streptococcus agalactiae*.

**Figure-3:**
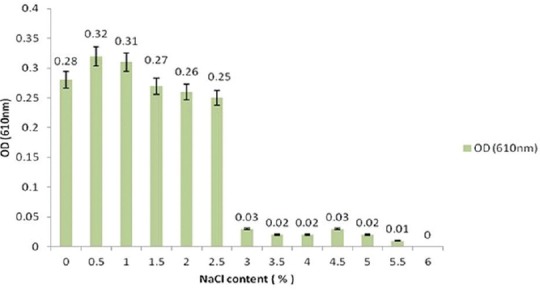
Effect of salinity on the growth of *Streptococcus agalactiae*.

*S. agalactiae* grew in ambient temperature ranging from 5 to 45°C, but the optimum growth temperature was 30°C (Figure-1). *S. agalactiae* grew at pH from 3 to 11, and the optimum pH was 7 (Figure-2). *S. agalactiae* grew at NaCl content in the medium from 0% to 5.5% (w/v) with an optimum of 0.5%. When NaCl content exceeded 5.5%, no growth was observed (Figure-3).

### 16S rRNA gene sequence analysis

**Figure-4:**
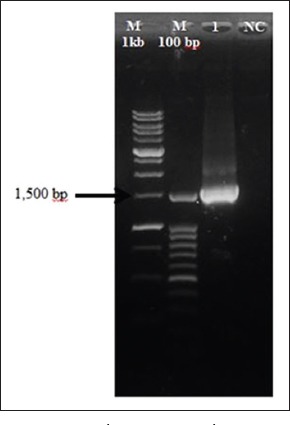
16S rRNA polymerase chain reaction product of *Streptococcus agalactiae* (Lane 1) amplified at 1500 bp with standard molecular weight marker (M 1 kb; M 100 bp) and negative control (NC).

The PCR product of the isolate was analyzed at the amplified fragments of approximately 1500 bp in size (Figure-4). The 16S rRNA sequence of the isolate was analyzed via BLAST network services. Sequence alignments with known sequences in the GenBank database showed that the brain isolate had high similarity (99%) to *S. agalactiae* isolated from China (Accession No. KU561093). The sequencing result of the isolate showed the sequence length of the PCR product was at 1201 bp and was deposited in GenBank (Accession No. KT869025).

### Antibiotic susceptibility test

The results are presented in Table-3 shows the sensitivity of the *S. agalactiae* strain tested, which exhibited resistance to neomycin and gentamicin; whereas was sensitive to 16 antimicrobial agents.

**Table-3:** Antimicrobial susceptibility test of *S. agalactiae* isolated from hybrid tilapia (*Oreochromis* spp.) via agar disc diffusion method.

Antibiotic	Disc potency (μg)	Bacteria (*S.agalactiae*)	Zone of inhibition*		

			R	I	S
Nitrofurantoin	50	I	19	20-25	26
Flumequine	30	S	15	16-18	21
Florfenicol	30	S	14	15-17	18
Amoxylin	25	S	13	14-17	18
Doxycycline	30	S	14	15-18	19
Oleandomycin	15	S	12	13-16	17
Tetracycline	30	S	14	15-18	19
Ampicillin	10	S	14	15-16	17
Lincomycin	15	S	14	15-20	21
Colistin sulfate	25	S	8	9-10	11
Oxolinic acid	2	S	14	15-17	18
Novobiocin	30	I	17	18-21	22
Spiramycin	100	S	12	13-15	16
Erythromycin	15	S	13	14-22	23
Fosfomycin	50	S	13	14-16	17
Neomycin	10	R	12	13-15	17
Gentamicin	10	R	12	13-14	15
Polymyxin B	300	S	8	9-11	12

* Susceptible (S), Intermediate susceptible (I), Resistant (R). *S. agalactiae*=*Streptococcus agalactiae*

### Histopathology

#### Clinical signs and postmortem lesions

**Figure-5:**
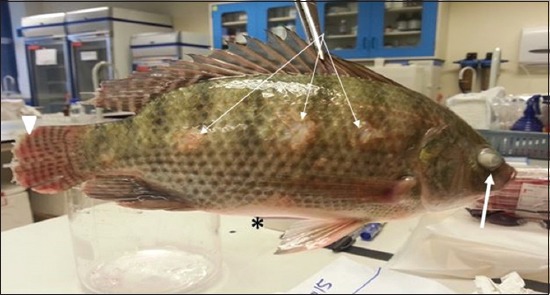
Hybrid tilapia (Oreochromis spp.) naturally infected by *Streptococcus agalactiae* showing ocular opacity (thick arrow), caudal fin hemorrhage and erosion (arrow head), erosion of the skin (thin arrow), and abdominal distention) (asterisk).

**Figure-6:**
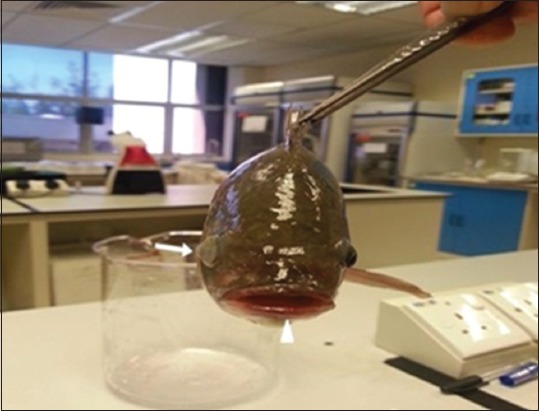
Hybrid tilapia (*Oreochromis* spp) naturally infected by *Streptococcus agalactiae* showing unilateral exophthalmia (thick arrow), and heavy hemorrhages on the lip and mouth (arrow head).

**Figure-7:**
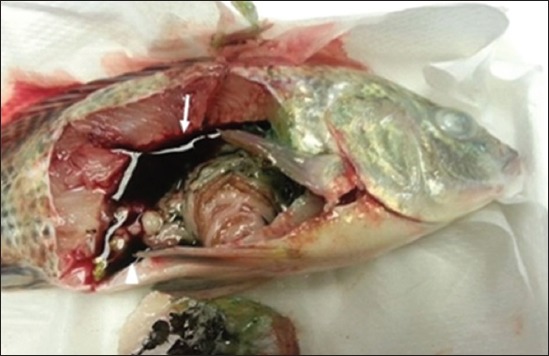
Hybrid tilapia (*Oreochromis* spp) naturally infected by *Streptococcus agalactiae* showing septicemic lesions with severe organ congestion, congestive kidneys (thick arrow), and bloody hemorrhagic ascetic fluids that were noticed on dissection (arrow head).

Most symptoms and clinical abnormalities including loss of appetite, orientation and erratic swimming, mortalities reached about 40% after 3^rd^ day from clinical abnormalities and sudden death. Postmortem examination of sacrificed fish revealed some fish exhibited hemorrhagic caudal fins, ophthalmic abnormalities including eye opacity and Some fish showed enlarged congested spleen and kidney. In addition, some fish showed heavy hemorrhages on the lip and mouth, severe congestion and congestive kidneys with bloody hemorrhagic ascetic fluids that were noticed on opening the fish as a black color fluid in abdomen (Figures-5-7).

### Histopathological examination results

**Figure-8:**
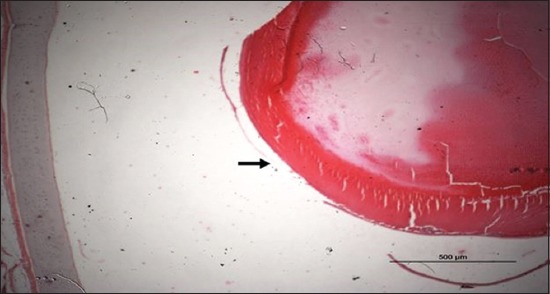
Eye section (H and E stain) of hybrid tilapia (*Oreochromis* spp.) naturally infected by *Streptococcus agalactiae* showing damage of lens capsule (arrow).

**Figure-9:**
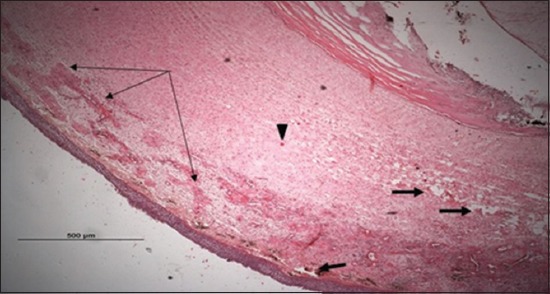
Eye section (H and E stain) of hybrid tilapia (*Oreochromis* spp.) naturally infected by *Streptococcus agalactiae* showing choroid edema (thick arrow), hyperemic choroid (thin arrow), and hemorrhage (arrow head).

**Figure-10:**
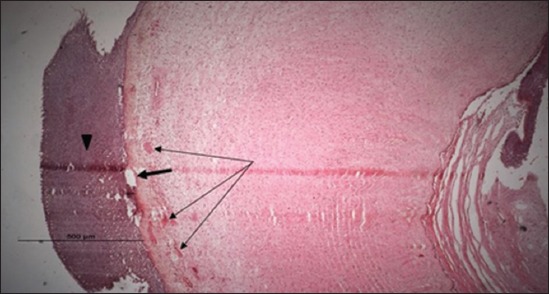
Eye section (H and E stain) of hybrid tilapia (Oreochromis spp.) naturally infected by *Streptococcus agalactiae* showing retina hyperplasia (arrow head), choroid hemorrhage (thin arrow), and choroid edema (thick arrow).

**Figure-11:**
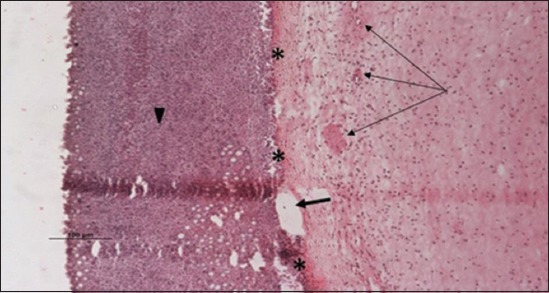
Eye section (H and E stain) of hybrid tilapia (*Oreochromis* spp.) naturally infected by *Streptococcus agalactiae* showing retina hyperplasia (arrow head), retina edema (thick arrow), choroid hemorrhage (thin arrow), and choroid edema (asterisk) with inflammatory cellular infiltration.

**Figure-12:**
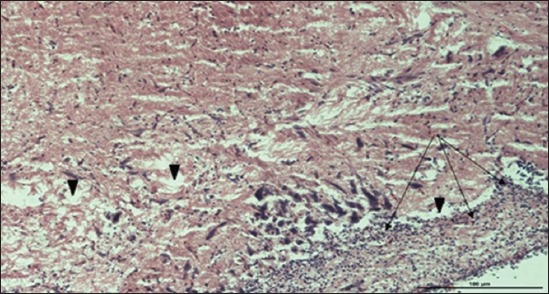
Brain section (H and E stain) of hybrid tilapia (*Oreochromis* spp.) naturally infected by *Streptococcus agalactiae* showing infiltration of inflammatory cell (thin arrow), perivascular and pericellular edema (head arrow).

**Figure-13:**
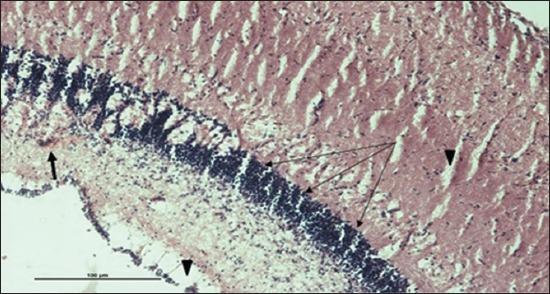
Brain section (H and E stain) of hybrid tilapia (*Oreochromis* spp.) naturally infected by *Streptococcus agalactiae* showing infiltration of inflammatory cell (thin arrow), perivascular and pericellular edema (head arrow), and hemorrhages (thick arrow) of the meninges.

**Figure-14:**
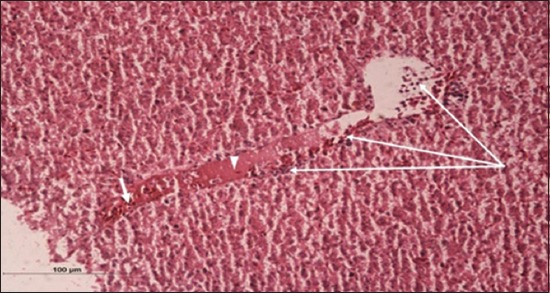
Liver section (H and E stain) of hybrid tilapia (*Oreochromis* spp.) naturally infected by *Streptococcus agalactiae* showing congestion of blood vessel (thick arrow), thrombosis of portal blood vessel (head arrow), and inflammatory cells infiltration (thin arrow).

**Figure-15:**
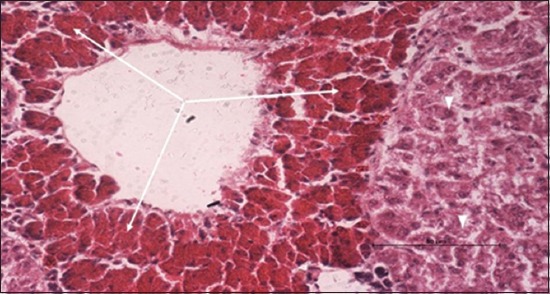
Liver section (H and E stain) of hybrid tilapia (*Oreochromis* spp.) naturally infected by *Streptococcus agalactiae* showing congestion of sinusoids and loss of hepatocytes (thin arrow), and vacuolar (fatty) degeneration of hepatocytes (head arrow).

**Figure-16:**
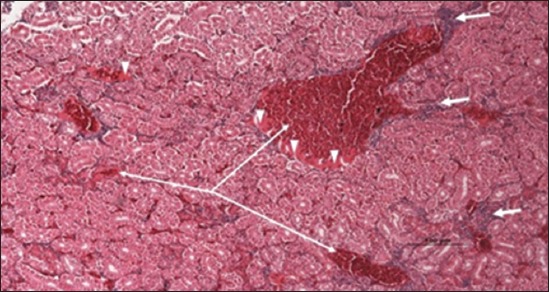
Kidney section (H and E stain) of hybrid tilapia (*Oreochromis* spp.) naturally infected by *Streptococcus agalactiae* showing hemorrhage (thin arrow), thrombosis (head arrow) in glomeruli and tubules, and infiltration of inflammatory cell (thick arrow).

**Figure-17:**
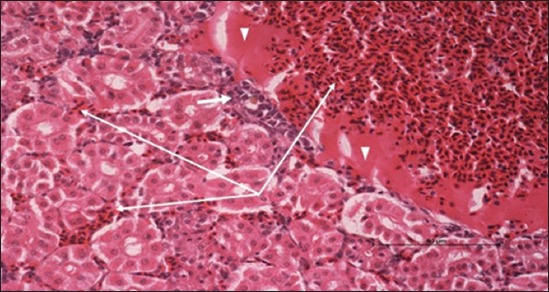
Kidney section (H and E stain) of hybrid tilapia (*Oreochromis* spp.) naturally infected by *Streptococcus agalactiae* showing hemorrhage (thin arrow), thrombosis (head arrow) and atrophy in hematopoietic tissue (thick arrow).

**Figure-18:**
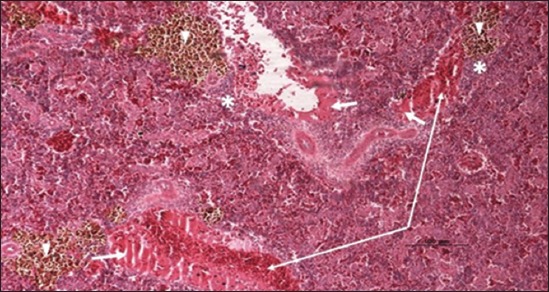
Spleen parenchyma section (H and E stain) of hybrid tilapia (*Oreochromis* spp.) naturally infected by *Streptococcus agalactiae* showing large thrombus in the splenic blood vessel (thick arrow), multifocal hemosiderin deposition (head arrow), congestion of blood vessels (thin arrow), and multifocal infiltration of macrophages (asterisk).

**Figure-19:**
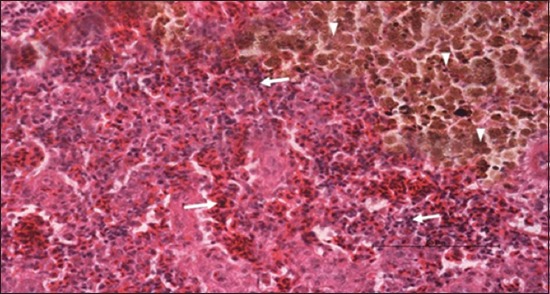
Spleen parenchyma section (H and E stain) of hybrid tilapia (*Oreochromis* spp.) naturally infected by *Streptococcus agalactiae* showing increased melanomacrophage cells (head arrow), and inflammatory cells infiltration (thick arrow).

Specimens were obtained from eye, brain, liver, kidney, and spleen of hybrid tilapia (*O. niloticus*) naturally infected by *S. agalactiae* revealed histological alterations was variable degrees of intensity. The eye lesions were characterized by damage of lens capsule and aqueous cavity. In addition to hyperplasia in retina, cellular infiltrate were observed in the choroid tissue with hyperemic lesions and inflammation. Periorbital edema was accompanied by inflammatory cellular infiltration and thrombosis (Figures-8-11). in regarsds to microscopic examination of brain specimens, the examined sections revealed congestion of meningeal vessels along with perivascular edema. Meningitis was an obvious sign characterized by thickening with congested blood vessels and inflammatory cells infiltration. Thus, inflammatory infiltration cell was observed in the cerebral cortex accompanied with variable degrees of neuronal degeneration were observed (Figures-12 and 13). Regarding the examined liver tissue specimens revealed congestion and thrombosis of portal blood vessel accompanied with hepatic sinusoids with marked vacuolar (fatty) degeneration change of hepatocytes. Furthermore, focal areas of inflammatory cells infiltration were noticed in hepatic tissue of fish (Figures-14 and 15). Concerning renal alterations, it was characterized by hemorrhage and thrombosis in glomeruli and tubules as well as infiltration of inflammatory cell and atrophy in hematopoietic tissue (Figures-16 and 17). In regards to the histopathological changes that were observed in the examined sections of spleen of the fish, it revealed vascular congestion surrounding with the inflammatory cells infiltration. On the other hand, thrombosis of the splenic blood vessels was clearly seen together with an increase in the melanomacrophage center in the splenic parenchyma (Figures-18 and 19). Histologically, the affected fish by *S. agalactiae* showed congestion, hemorrhages and inflammation in several internal organs, particularly the liver, heart, spleen, kidney, eyes, and brain as systemic phase of the septicemia disease.

## Discussion

Tilapia (*O. niloticus*) is a key species being cultured in Malaysia and other South East Asian countries that are high-priced and popular. In the last decades, many cultured tilapia species have become susceptible to Streptococcosis, an aggressive disease constantly threatening the sustainability of the global tilapia industry [3,7,12,23]. This disease is characterized grossly by hemorrhages and hyperemia, especially of the fins, unilateral exophthalmos with intraocular hemorrhages, kidney enlargement, as well as high mortality. The aim of this study was to use molecular approaches to investigate the relative contributions of composition of hybrid tilapia (*O. niloticus*) naturally infected by *S. agalactiae* with histopathological emphasis on lesions in eye, brain, kidney, liver, and spleen. Histopathological examination provides a methodological platform to determine the effect of bacteria in various organs [1]. In addition, using PCR methods have shown to be very reliable in the detection of *S. agalactiae* as well as a vast reduction in time in comparison with culture methods [24]. Most new research depends on biochemical and molecular methods for identifying and characterizing bacteria. This study was done using standardized set of biochemical identification and confirmed by molecular approaches (16S rRNA gene PCR) to the isolated bacteria. Hence, we have not focused on the pathogenicity of these bacteria in this study.

In this study, *S. agalactiae* were isolated from the eye of diseased fish hybrid tilapia (*O. niloticus*). *S. agalactiae*, which is considered the main etiological agent of clinical diseases in healthy fish, is a pathogen of interest to several researchers whose works all involve the pathogenicity of *S. agalactiae* in causing disease in fish [8,12,25,26]. The hybrid tilapia (*O. niloticus*) naturally infected by *S. agalactiae* revealed several clinical manifestations consistent with the disease including; erratic swimming, corneal opacity, hemorrhage of the fin and internal organs. Histopathological abnormalities observed in the brain, kidney and eye tissues are suggestive of septicemia as observed in natural and experimental Streptococcal infection [5,8,17,23,27,28]. Meanwhile, Yiagnisis and Athanassopoulou [29] stated that clinical manifestations caused by any pathogen are dependent upon certain factors including the type of host, age of organism, and the stage of disease (acute or chronic). Furthermore, in several cases, there can be no correlation found between internal and external damages. There are cases in which systemic diseases with high mortality rates caused extensive internal damage to infected fish, but often appearing healthy. On the other hand, several diseases with a relatively low mortality are capable of causing significant damage such as ulcers, necrosis, and exophthalmos, rendering those fish unfit for consumer sales. The gross pathological abnormalities were typical manifestations of acute septicemia, as in agreement with Netto *et al*. [25] and Mian *et al*. [30] who concluded that *S. agalactiae* is characterized by septicemia and meningoencephalitis in fish. Furthermore, fish bacterial infections can arise as a septicemia where bacteria and its extracellular products exist in the circulatory system disrupting fish physiological functions and induce variety of pathological alterations that may lead to death. Thus, sudden death may be due to acute systemic infection of natural infection by *S. agalactiae* [12]. Studies of eye lesions are usually commonly observed in most of systemic bacterial diseases. Hence, exophthalmia occurred by corneal opacity in fish infected by *S. agalactiae* and histopathological examination showed lesions involving periorbital tissue and choroid in addition to moderate vascular hemorrhage. Our results were in accordance with many researchers who reported exophthalmia in tilapia infected with *Streptococcus* spp., with lesions being discovered in periorbital and choroid tissue [5,31,32]. Histopathological studies of brain tissue specimens show meningeal thickening and inflammatory cell infiltration in the brain tissue, indicating meningitis, accompanied by hemorrhage similar finding reported in various study [7,10,33-35]. These results demonstrated that macrophages can possibly act as a vehicle for *S. agalactiae*, allowing it to cross the blood-brain barrier and gain access to the central nervous system, thereby becoming disseminated throughout the organism’s organ systems initiating bacterial septicemia [36]. The pathological manifestation in the eyes and brain tissue of naturally infected fish correlates with the clinical behavioral abnormalities of erratic pattern of swimming, orientation abnormalities. These results support the theories by several authors who state that *S. agalactiae* is neurotropic [7,28,32,37,38].

The histopathological changes observed in liver revealed marked degeneration of the hepatocytes and congestion in the sinusoid due to the inability of the liver to detoxify the foreign body and resulting in liver dysfunction and eventually death [39]. In addition, *S. agalactiae* infection in tilapia liver demonstrated vacuolation of hepatocytes with fatty change in response to the reduced blood flow due to congestion of blood vessel and thrombosis of portal blood vessel [32,35,37]. Histological analysis of the kidney in this study was characterized by severe hemorrhage and thrombosis accompanied with infiltration of inflammatory cell, and severe atrophy in hemopoietic tissue similar to the experimental infection in red tilapia (*Oreochromis* sp.) with *S. agalactiae* [12,23]. The histopathological examination of spleen tissues revealed red pulp degeneration, splenic capillary congestion, and focal hemorrhages. Furthermore, large areas of hemosiderin deposits and melanomacrophage center hypertrophy as similarly reported by Filho *et al*. [32]. The proliferation of the hematopoietic in spleen tissue and activation of the main phagocytic cells, melanomacrophages, may be due to the role of bacteria and its toxins to stimulate the immune response in infected fish [40]. The lesions in the hemopoietic tissue including kidneys and spleen indicated that the *S. agalactiae* infection may suppress the immune system and increasing host susceptibility to infection with other microorganisms.

Resistance to generic antibiotics is a developing issue among *Streptococcus* spp. [41]. The degree of susceptibility of the *S. agalactiae* toward 18 different types of antibiotics in this study revealed *S. agalactiae* to be resistant to neomycin and gentamicin, which is in accordance with similar result reported by Abuseliana *et al*. [7] in fish infected with *S. agalactiae* isolated from red tilapia in Malaysia and to Geng *et al*. [42] who also described resistance of *S. agalactiae* to gentamicin. Neomycin and gentamicin, which are wide spectrum aminoglycoside antibiotics, work by establishing a binding process to the receptors found on the 30S ribosomal subunit of bacteria; therefore, preventing the initiation complex between bacterial messenger RNA and the ribosomal subunit leasing to misreading of the bacterial DNA and formation of nonfunctional proteins thereby affecting its survival.

Several environmental conditions existing during disease progress have been thought to be causative factors in influencing fish to infections such stressors are most commonly associated with high water temperature [6,13], susceptibility of tilapia [7,12], pathogenicity of *S. agalactiae* [7], and the high-density fish culture would all increase the susceptibly of fish to *S. agalactiae* infections [43].

Hence, the detection of the pathogens described in this study with the emphasis on transmission risk to other farmed fish and the establishment of procedures to improve quarantine and pathogen screening is needed. In addition, it is recommended that generic antibiotics be used with caution in the treatment of fish infections unless antibiotic sensitivity testing can be done. Finally, it is strongly suggested that both PCR assay methods and culture methods be routinely utilized for the accurate detection of *S. agalactiae* in tilapia.

## Conclusion

The findings of this study reveal the need to raise future epidemiological and pathogenesis studies contributing to a greater understanding of the behavior of the bacteria in a population and within the host, in addition to design control strategies. Because most *S. agalactiae* isolates displayed resistance to at least two antibiotics; therefore, molecular procedures should be utilized in determining the antibiotic resistance profiles of the isolates in order to properly control infections. Besides, this study has established that the histopathological changes of Tilapia could be helpful in the diagnosis of the pathological and physiological standings in Tilapia culture.

## Authors’ Contributions

AAL conducted the design of the experiment. AAL, MAA, MH, SMS, ASD, WW, and AJ involved in the field and laboratory work for data collection. WNWI and ASA, contributed in the confirmation of *Streptococcus agalactiae* by 16s rRNA gene sequencing. AAL and WNWI involved in data analysis. MN deposited the accession number of *Streptococcus agalactiae* in GenBank. AAL, WNWI, MNaj with the help of AJ analyzed and reviewed the manuscript. All authors read and approved the final manuscript.
